# Arabic speech recognition model using Baidu's deep and cluster learning

**DOI:** 10.3389/frai.2025.1639147

**Published:** 2025-09-04

**Authors:** Fawaz S. Al-Anzi, Bibin Shalini Sundaram Thankaleela

**Affiliations:** Department of Computer Engineering, College of Engineering and Petroleum, Kuwait University, Kuwait

**Keywords:** clustering, language model, acoustic model, Baidus deep speech, RNN, deep learning

## Abstract

This study involves extracting the spectrum from the Arabic raw, unlabeled audio signal and producing Mel-frequency cepstral coefficients (MFCCs). The clustering algorithm groups the retrieved MFCCs with analogous features. The K-means clustering technique played a crucial role in our research, enabling the unsupervised categorization of unlabeled Arabic audio data. Employing K-means on the extracted MFCC features allowed us to classify acoustically similar segments into distinct groups without prior knowledge of their characteristics. This initial phase was crucial for understanding the inherent diversity in our diverse sampled dataset. Dynamic Time Warping (DTW) and Euclidean Distance are utilized for illustration. Classification algorithms such as Decision Tree, eXtreme Gradient Boosting (XGBoost), K-Nearest Neighbors (KNN), and Random Forest are used to classify the various classes obtained based on clustering. This study also demonstrates the efficacy of Mozilla's Deep Speech framework for Arabic speech recognition. The core component of deep speech is its neural network architecture, which consists of multiple layers of Recurrent Neural Networks (RNNs). It strives to comprehend the intricate patterns and interactions between spoken sounds and their corresponding textual representations. The clustered labeled Arabic audio dataset, along with transcripts and Arabic Alphabets, is used as input to Baidu's Deep Speech model for training and testing purposes. PyCharm, in conjunction with Python 3.6, is used to build a Dockerfile. Creating, editing, and managing Dockerfiles within PyCharm's IDE is simplified by its functionality and integrated environment. Deep speech provides an eminent Arabic speech recognition quality with reduced loss, word error rate (WER), and character error rate (CER). Baidu's Deep Speech intends to achieve high performance in both end-to-end and isolated speech recognition with good precision and a low word rate and character error rate in a reasonable amount of time. The suggested strategy yielded a loss of 276.147, a word error rate of 0.3720, and a character error rate of 0.0568. This technique increases the accuracy of Arabic automatic speech recognition (ASR).

## 1 Introduction

Speech acts as a gateway in communicating our ideas through different vocal sounds and is a powerful tool that shapes our world. The study of speech signals and the techniques used to process them is known as speech processing. Modern automatic speech recognition (ASR) systems replace the conventional human–machine interface in various commercial applications. Through the application of linguistics and computer science, ASR systems can interpret spoken words and translate them into text. This enables voice-activated device interaction, message dictation, and generation of transcripts from recordings. Recent developments in artificial intelligence (AI), particularly natural language processing (NLP), have focused on using AI applications for ASR. Researchers have investigated morphological analysis, resource building, and machine translation for the Arabic language. Speech and language disorders are a side effect of many diseases, and devices like the Servox Digital Electro-Larynx (EL) can generate quasi-clear voices for people with disorders (Mohammed Ameen and Abdulrahman Kadhim, 2023). The respiratory, phonatory, and articulatory end organs are all involved in the intricate neurological process of speech (Musikic et al., 2025). Acoustic media and background noise can disrupt and interfere with speech communication. Vocalization system damage can affect the efficiency of voice recognition and voice clarity (Liu et al., 2025). ASR is useful in many domains, including the development of accessible applications to transforming human–machine communication. Speech recognition automatically identifies and translates a person's spoken words based on the data available in a speech waveform and its historical data feed. The evolution of deep learning has changed the ASR landscape in conjunction with Recurrent Neural Network (RNNs), deep neural networks (DNNs), and convolutional neural networks (CNNs). Deep neural networks are multilayered artificial intelligence that learns from data. They are inspired by the structure of the human brain, and these layers enable them to handle challenging issues. Deep neural networks, which have been trained on enormous datasets, modify their internal connections to identify patterns and carry out tasks such as speech translation and image recognition. The ability of CNNs to extract intricate patterns from audio input has been inspiring. Baidu's Deep Voice enhances voice recognition precision in noisy situations, as well as in far-field and reverberant conditions (Ahmed and Ghabayen, 2017; Masterson, 2015). MFCCs effectively decipher sound content in speech and audio processing. The MEL scale considers how our ears interpret pitch and frequencies with similar sounds. Applications such as speech recognition systems can interpret speech data by evaluating MFCCs. A clustering algorithm is a specific set of instructions that tells a computer how to automatically group data points into clusters. The study addresses the issue of unlabeled Arabic audio data by applying an unsupervised clustering algorithm to analyze and structure the corpus, uncovering acoustic patterns, speaker variabilities, and environmental conditions. These insights inform effective data handling strategies and the training of Arabic Deep Speech ASR models. These algorithms are used in unsupervised learning, where the data does not have predefined labels. There are many clustering algorithms, but one of the popular popular ones is K-means. Algorithms such as Hierarchical clustering, Mean shift clustering, Gaussian mixture model, Affinity propagation, and K-means clustering are widely available to group different patterns of MFCCs (Al-Anzi and Shalini, 2024).

The primary objective of this study is to develop an ASR system that automatically transcribes spoken utterances into a textual format. Our approach utilized a database consisting of Arabic audio recordings, which encompassed news broadcasts, public speeches, and various general recordings of individuals. The primary objective of our study is to extract the Mel-frequency coefficients necessary for ASR from the unlabeled Arabic audio dataset. We employed a clustering approach, with the clusters organized according to the KNN algorithm to label the collected MFCCs. The retrieved MFCCs are categorized according to their auditory characteristics. We have utilized Baidu's Deep Speech model to transcribe spoken language into text. The input given to the model is our clustered Arabic audio dataset along with its transcribe and alphabet. We also assessed the word error rate (WER) and character error rate (CER) of the transcribed results from the audio datasets. We have labeled the clustered dataset using a speech recognition pretrained model from the klaam library, categorizing it as Modern Standard Arabic (MSA), Egyptian Arabic (EGY), and Gulf Arabic (GLF) based on dialects. We have trained the model using different machine learning algorithms to categorize the dialects and assess accuracy, loss, and evaluation metrics for the clustered results.

The subsequent sections of the article are structured as follows: A concise literature overview encompassing ASR, diverse languages and accents in ASR, end-to-end speech processing, and the deep learning architectures that facilitate speech recognition, concluding with a clearly defined research gap, along with the methodologies and materials. Includes fundamental architecture, data collection, data analysis, MFCC analysis, clustering of MFCC characteristics, classification, performance evaluation, findings, debates, conclusion, and future scope.

## 2 Literature review

The study by Ahmed and Ghabayen (2017) proposes three methods to improve Arabic automatic speech recognition. They are listed in the following order: utilizing a Decision Tree to generate alternative pronunciations, modifying a native acoustic model with a different native model, and text processing to improve the language model. By employing these methods, the word error rate was reduced. The methodology of the paper showed how deep speech recognition models can integrate over time with long, adjustable windows (Ahmed and Ghabayen, 2017).

### 2.1 Automatic speech recognition

In the study by Keshishian et al. (2021), ASR aims to enable computers to identify and interpret human speech as accurately as possible. Many techniques can be used to implement speech recognition models. The author utilized one of the newest techniques for speech recognition, which employs neural networks with deep learning. An overview of the research conducted on Arabic voice recognition is given in the paper by Wlgihab et al. It also sheds some light on the facilities and toolkits available for Arabic voice recognition system development (Algihab et al., 2019). A vast array of products has been developed that efficiently leverage ASR to enable communication between humans and machines by Karpagavalli and Chandra et al. Speech recognition applications exhibit reduced performance in the presence of reverberation or minimal background noise (Karpagavalli and Chandra, 2016). Both acoustic and text transcriptions are used during the entire training process of ASR neural network systems.

The study by Belinkov et al. compares phonemes and graphemes along with different articulatory properties to evaluate the representation quality across a range of classification tasks. The study analyzes three datasets and two languages, Arabic and English, and demonstrates how consistently different features are represented across deep neural network covers (Belinkov et al., 2019). The purpose of the study by Abdul et al. is to discuss the applications of the MFCC as well as certain problems with its calculation and how they affect the model's performance (Abdul and Al-Talabani, 2022). An enhanced Mel-frequency cepstral coefficients (MFCC) feature for unsupervised marine target clustering is presented in the research. It exhibits a high success rate for multitarget or depth-target clustering as well as strong anti-interference capabilities (Yang and Zhou, 2018). The Short-Time Fan-Chirp Transform (FChT), a novel technique for time-frequency analysis of speech signals, is presented in this study (Képesi and Weruaga, 2006). It enhances spectral and time-frequency representation, making it appropriate for filtering applications. Taking contextual considerations into account, this method examines speech processing to quantify controllable speech features across a variety of talker populations, noise levels, competing speakers, and the channel through which it is conveyed (Pitton et al., 1996).

The study by Abushariah et al. gave a framework for designing a speaker-independent automatic Arabic speech recognition system using a phonetically rich speech corpus. The system uses Carnegie Mellon University's Sphinx tools and Cambridge HTK tools and uses three-emitting state Hidden Markov Models for tri-phone-based acoustic models. The system achieved word recognition accuracy of 92.67 and 93.88% for similar speakers with different sentences, and a Word Error Rate of 11.27 and 10.07% with and without diacritical marks (Abushariah et al., 2012). A simple word decomposition algorithm presented by Afify et al. requires a text corpus and affix list, improving WER by 10% in Iraqi Arabic ASR. The algorithm also reduces WER by 13% relative (Afify et al., 2006). The research presented by Ali Ahamed et al. shows a novel methodology for assessing ASR in languages lacking a standardized orthographic system. The authors solicited five distinct users to transcribe speech segments, subsequently integrating the alignments from numerous references and presenting a revised WER. The findings indicated an average WER of 71.4 and 80.1%, respectively.

### 2.2 Different languages, ascent speech recognition

To build high-performing recognizers for two radically different languages, such as Mandarin and English, the authors Amodei et al. looked into a variety of network topologies and found a few helpful techniques, such as look-ahead convolution for unidirectional models, and enhanced numerical optimization using SortaGrad and Batch Normalization (Amodei et al., 2016). In the study by Nahid et al., they investigated the capacity of the DeepSpeech network to recognize unique Bengali speech samples. Recurrent Long Short-Term Memory (LSTM) layers form the foundation of this network, which models internal phoneme representations. At the bottom, convolutional layers are added, which removes the requirement to assume anything about internal phoneme alignment. The model was trained using a connectionist temporal classification (CTC) loss task, and the transcript was generated by casting a beam search decoder. On the Bengali real number speech dataset, the developed method produced a lower word error rate and a character error rate (Nahid et al., 2019).

In the study by Priyank Dubey (2023), they discussed that the transcription of spoken speech can be extracted from the waveform using ASR. Mozilla Deep Speech is among the most recent, according to Baidu's Deep Speech research report. Through end-to-end deep learning, the state-of-the-art deep voice recognition system was developed. A properly optimized RNN is used with several Graphical Processing Units (GPUs). Its generalizability to other English accents is limited because American English accents make up the majority of the datasets used in this training. In this study, researchers used the most recent Deep Voice model, Deep Speech-0.9.3, to create an Indian-English speech recognition system from beginning to end for dialects. In the study by Xu et al. (2020), the focus of the research was on a real-time German speech-to-text system that was constructed using numerous German language datasets. Researchers in this study optimized DeepSpeech for teaching a current German speech-to-text prototype by combining multiple German datasets. Moreover, they achieved strong WER rates. The model discussed in the study by Ai-Zaro et al. produces the WER/PER of 3.11 and 6.18% (Al-Zaro et al., 2025).

Literature (Iakushkin et al., 2018) explains how a voice recognition system for the Russian language is made using DeepSpeech. The foundation was the Mozilla Corporation's DeepSpeech English implementation, which is available as open-source software. The system was trained in a containerized environment using Docker technology. A dataset of Russian literary audio recordings made available on voxforge.com was used, and the best WER was 18%. A study by Messaoudi et al. (2021) proposes an end-to-end method for building Tunisian language communication systems based on deep learning. The paired text-speech dataset in the Tunisian dialect created for this proposal is called “TunSpeech.” Furthermore, the current Modern Standard Arabic (MSA) speech data were combined with dialectal Tunisian speech data to lower the Out-of-Vocabulary rate.

### 2.3 End-to-end speech processing

Research (Kim et al., 2017) offers a novel end-to-end speech recognition method that leverages a hybrid CTC-attention model within a multitask learning framework to boost resilience and accelerate convergence, thereby reducing the alignment issue. An experiment using the WSJ and CHiME-4 tasks demonstrates its superiority over the CTC and attention-based encoder-decoder baselines, yielding 5.4–14.6% relative improvements in CER. The study by Agarwal and Zesch (2020) utilizes a shared task on SwissText/KONVENS for a speech-to-text system. A neural network is trained end to end, using Mozilla DeepSpeech as its foundation. Data augmentation, post-processing, and transfer learning from standard English and German were utilized. The WER generated by the system is 58.9%.

### 2.4 Speech recognition using deep learning

In the study by Nedal Turab (2014)), a neural network technique was used to address phoneme recognition. Gaussian low-pass filtering produced improved voice signal quality and reduced noise, which was then used to train a neural network for system training. Study (Alrumiah and Al-Shargabi, 2023) tackles the important task of identifying classic Arabic speech for the 1.9 billion Muslims who recite the Quran. It proposes a model based on Deep Neural Networks (DNNs). With a 19.43% word error rate and a 3.51% character error rate, RNN-CTC outperformed the other models following its training on a 100-h dataset of Quran recordings. CNN was used to further reduce the word error rate. Paper (Alsayadi et al., 2021) presents Arabic diacritical mark-based ASR systems. To create a trustworthy and accurate Arabic ASR, a study by Alsayadi et al. looks at the application of cutting-edge end-to-end deep learning techniques. The acoustic characteristics used in these methods are the log Mel-Scale Filter Bank energies and the Mel-frequency cepstral coefficients. Enhancing discretized Arabic ASR is possible with CNN-LSTM and a new CTC-based ASR. When it comes to Arabic voice recognition, CNN-LSTM with a consideration basis outperforms both traditional ASR and the Joint CTC-attention ASR context (Alsayadi et al., 2021). The research by Ullah et al. utilized Arabic image datasets that have been gathered, consisting of 2,000 Arabic digit records and 900 Arabic phrase records from 24 native speakers. VGG-19 is a deep convolutional neural network with 19 weight layers and is used in this study to extract visual characteristics. Two different approaches, namely, the batch-normalized VGG-19 base model and the standard VGG-19 base model, are presented in the study. The test dataset produces the accuracy of 93% digit and phrase recognition, 97% phrase recognition, and 94%-digit acknowledgment rates (Ullah et al., 2022).

Nagamine et al. analyze a sigmoid DNN trained for a phoneme recognition task to characterize different aspects of the non-linear changes that occur in hidden layers. The more separable phone instances are handled by deeper layers of the network through a non-linear feature space transformation. The study describes how a deep neural network model learns by transforming the feature space in a non-uniform way through repeated non-linear transformations (Nagamine et al., 2016). In the study by Hori et al. (2018), researchers investigate the impact of word-based RNN philological mockups language models (RNN-LMs) on end-to-end ASR performance. It includes a novel word-based RNN-LM which allows decoding with only word-based. Low WER is achieved by the proposed model for the WSJ Eval'92 test set. In the study by Dendani et al. (2020), the representational characteristics of a DNN trained for phoneme recognition were described. In the first hidden layer, node selectivity to specific articulation styles and locations appeared, and in the deeper layers, this selectivity became more pronounced. In the study by Dendani et al. (2020), ASR is implemented using a Deep Auto Encoder (DAE). The results showed that the enhanced speech's accuracy was about 3.17 times better than the accuracy estimated before. Recent models and algorithms, such as Mozilla Deep Speech, have been developed, but their generalizability is limited due to their use of American–English accent datasets (Priyank Dubey, 2023). The study by Srivathshan et al. proposes a hybrid Active Noise Cancellation (ANC) system that combines Secondary-Path Filtered Active Noise Control (SF-ANC) and a Fuzzy Adaptive Neuro-Fuzzy Inference System (FxANFIS) to improve noise reduction performance (Srivathshan et al., 2025).

### 2.5 Research gap

We haven't found any specific results from my more targeted searches for studies that directly combine Baidu's Deep Speech with cluster learning for Arabic speech recognition. Research on combining Baidu's Deep Speech and cluster learning for Arabic speech recognition has not yielded specific results, suggesting a lack of extensive exploration. However, studies using Deep Speech and cluster learning techniques have revealed challenges like language complexity and data limitations. This supports the hypothesis that this specific combination may not yet have been thoroughly investigated by researchers.

## 3 Methods and materials

The unlabeled Arabic audio dataset, along with the alphabet, is applied in the proposed work. The auditory data are converted and then hooked onto a sequence of probabilities spanning the characters in the alphabet. Second, this sequence of possibilities gives rise to a cast of characters. The first and second steps are made possible by a Deep Neural Network and an n-gram language model, respectively. The n-gram language model is trained on a text corpus, and the neural network is trained on corresponding text transcripts and audio files. To predict text from speech and prior text, respectively, both the language model and the neural model receive training. Generating (MFCC, Analog to Digital Conversion, Framing, Windowing, Discrete Fourier Transform conversion, Mel-Filter Banks Wrapping Frequency, Converting Mel Filter Banks to Log, Executing Discrete Cosine Transform, the Resultant MFCC Acoustic Model generation, Language Model creation, and Decoding algorithm with deep speech are the fundamental techniques employed in this system. They are all converted to a WAV setup and given a monaural aural canal with a sampling rate of 16,000 Hz and a depth of 16 bits for each value to allow our deep speech pipeline to read all audio clips.

Our unlabeled Arabic audio dataset was subjected to a clustering technique and was mainly used in the pre-processing and data interpretation phases. Since our original dataset was completely unlabeled, we used clustering to characterize acoustic diversity, which involves identifying distinct acoustic groups. The results obtained are manually tested against the transcribed text data. The clustering algorithm enables us to find hidden structures in the data by grouping the MFCC features. The MFCCs are derived from the available Arabic Audio datasets, which are further clustered based on their similar features using clustering algorithms. Machine learning algorithms are further introduced to classify the clusters. The combination of MFCC extraction, clustering, and classification provides an effective framework for extracting insightful information from Arabic speech data. Speech analysis tasks are a good fit for MFCCs because they capture the aspects of speech that are perceptible to humans. ASR allows voice-activated computer communication for individuals with physical disabilities. Mozilla's Deep Speech is one of the well-known ASR systems widely accepted and has shown remarkable progress in multiple languages, including Arabic. Baidu's Deep Speech framework is an open-source ASR system that converts spoken words into written language. This speech-to-text technology uses deep learning algorithms to translate spoken language into written text. Acoustic models, language, speech coherence, and performance evaluation are a few components of speech recognition models.

### 3.1 Methodology

**Figure 1:**
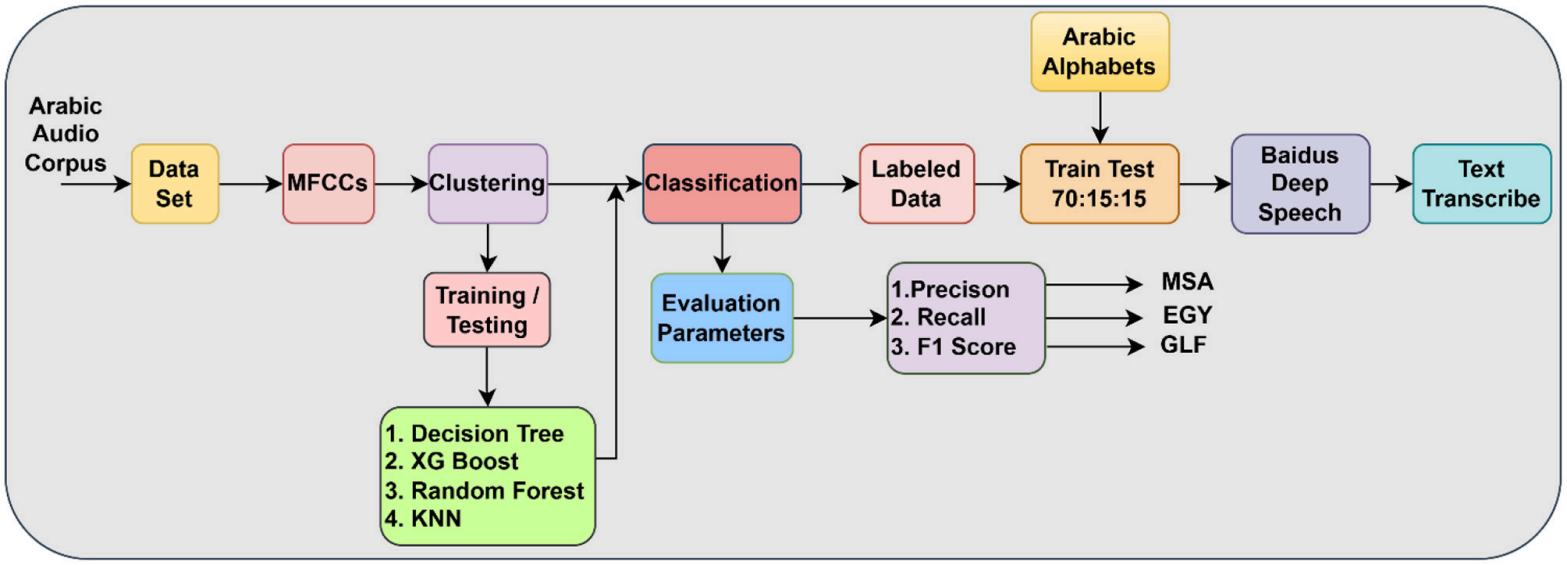
Methodology diagram with clustering and Baidu's deep speech.

Figure 1 depicts a detailed pipeline for processing Arabic audio data, incorporating both unsupervised and supervised machine learning methods alongside a deep learning model for transcription. The method commences with an Arabic Audio Corpus, which is subsequently input into a dataset preparation phase. MFCCs are recovered from this dataset, functioning as resilient acoustic characteristics. The characteristics subsequently undergo Clustering, wherein an unsupervised algorithm, presumably K-means, categorizes the audio segments according to their acoustic similarities. The speech recognition pretrained model by the klaam library labeled the clustered output as MSA, EGY, and GLF. The efficacy of the classification models is evaluated by metrics such as Precision, Recall, and F1-Score, with distinct results highlighting an emphasis on dialectal performance. The result of this clustering phase initiates a Training/Testing phase for traditional machine learning models, such as Decision Trees, XGBoost, Random Forest, and KNN, employed for a Classification task, presumably aimed at categorizing audio segments based on insights derived from the clustering. The classification outcomes, combined with the “Arabic Alphabets” input, facilitate the generation of labeled data, which is thereafter divided into 70% for training, 15% for testing, and 15% for validation. These annotated data are essential for training Baidu's DeepSpeech model, the fundamental element responsible for the Text Transcribe job, which converts Arabic audio into text. This integrated architecture exemplifies a multifaceted strategy for Arabic speech processing, amalgamating feature engineering, unsupervised learning, conventional classification, and deep learning to provide a holistic solution.

### 3.2 Architecture of the speech recognition system

**Figure 2:**
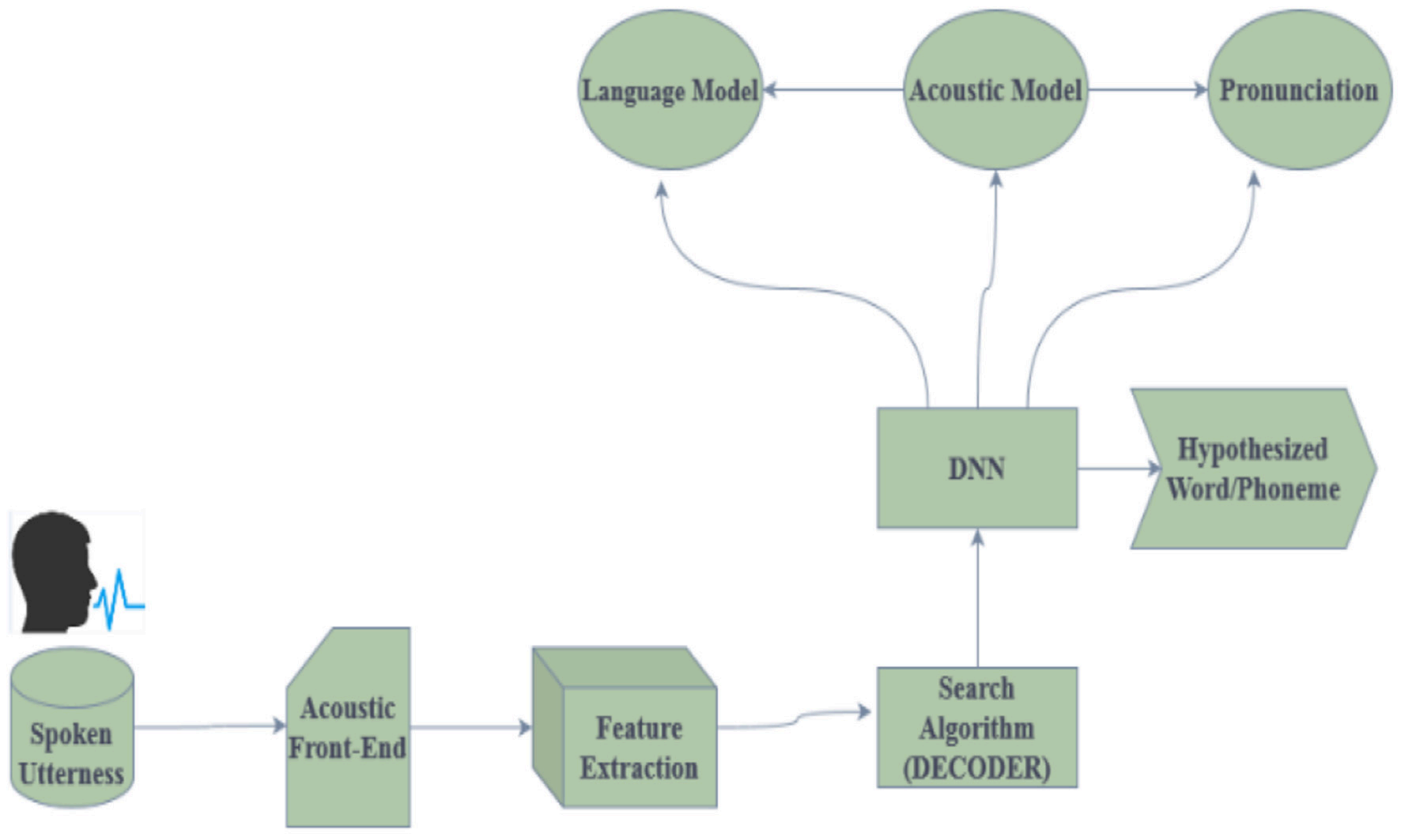
Architecture of the speech recognition system.

Figure 2 shows the architecture of the Speech Recognition System. Deep neural networks are used in speech recognition to translate spoken words into written text. To extract significant acoustic properties, the spoken utterances are first preprocessed. The following steps correspond to the preprocessing, feature extraction phases, decoder, and model creation. The preprocessing block performs various operations on the speech signal, such as noise reduction and silence removal. After the noise reduction, the background noise gets removed. There will not be any background noise in the spoken signal after the preprocessing phase. Scaling the voice signal to a standard magnitude is known as normalization. The speech stream is divided into shorter segments through framing, and these segments typically last 20–30 ms.

The process of extracting information from each voice signal frame is known as feature extraction. The acoustic properties of the voice signal are represented by these features. These characteristics are then applied to a series of models: an audio model forecasts the phoneme sequence, and a dialectal prototypical model uses the analysis of the previous word to predict the next. A decoder transforms the sequence into a string of words, enabling accurate speech-to-text conversion. This process uses a pronunciation dictionary to ensure accurate translation and proper word pronunciation. The retrieved features in the acoustic model, a statistical model, represent a set of phonemes. The language model is a numerical model that forecasts the next verse in a series based on the verses that have already been spoken. The decoder needs to convert the sequence of phonemes from the acoustic model into a word order. The last block in the diagram represents the word sequences that have been transcribed. A string of words represents spoken speech. Because DNNs can identify complex patterns in data, they are well-suited for voice recognition tasks.

#### 3.2.1 Probability theory for speech recognition

An ASR system's main objective is to infer the acoustic input O in Equation 1, the most likely discrete symbol sequence among all valid sequences in the language L (Rabiner and Juang, 1993).


(1) $$\begin{array}{l} O=o_{1},o_{2},o_{3}\ldots .o_{t} \end{array}$$

The symbol sequence to be recognized is N, given in Equation 2:


(2) $$\begin{array}{l} N=n_{1},n_{2},n_{3}\ldots .n_{n} \end{array}$$

The fundamental ASR system goal and the probability are given in Equations 3, 4.


(3) $$\begin{array}{l} W=\text{argmax}P\left(W/O\right) \end{array}$$

(4) $$\begin{array}{l} P\left(W/O\right)=\frac{P\left(O/W\right)}{P\left(O\right)}\;P\left(W\right) \end{array}$$

### 3.3 Data collection

The Arabic audio dataset is our in-house dataset, which contains 4,071 audio samples from various fields, such as security and justice, Economy, Education, Health, Technology, and Sports. Each heading of data is subdivided into three levels of datasets, such as first, second, and third sets. Deep speech requires mono-channel audio files in WAV format with a sampling rate of 16 kHz and an encoding of 2 bytes per sample for all WAV files, so ensuring consistency in audio quality and format. This collection is categorized by speech type, comprising 733 spontaneous voice files and 588 read speech files, providing a varied representation of natural and controlled verbal expressions. The text linked to these audio recordings has an average length of 93.0 characters, reflecting a moderate complexity and vocabulary range within the collection. Ten to twenty-second passes are available between each voice sample. The more closely we match this, the longer or shorter the model will be. The alphabet.txt file contains a transcription of every character from the given voice clip. From the audio voice clip, all punctuation has been removed, including quotation marks, dashes, and other marks. Three sets of data were separated: test, validation, and training. Diacritical marks are used to show proper pronunciation or to provide phonetic guidance because the standard Arabic script does not provide enough information about pronunciation. Since deep speech operates at the character level, the inclusion of these representations influenced the generation of the acoustic model. Prediction possibilities rise based on the number of letters.

### 3.4 Data analysis

We have used a sample rate of 1,600 Hz for each audio data. The encoding of each wave file is 2 bytes per sample. Likely, spontaneous speech is used for our analysis. The number of spontaneous speech files is 733, and the number of speech files read is 588. The total number of training files is 1,321. The average text length is 93.0.

#### 3.4.1 Silence removal

**Figure 3:**
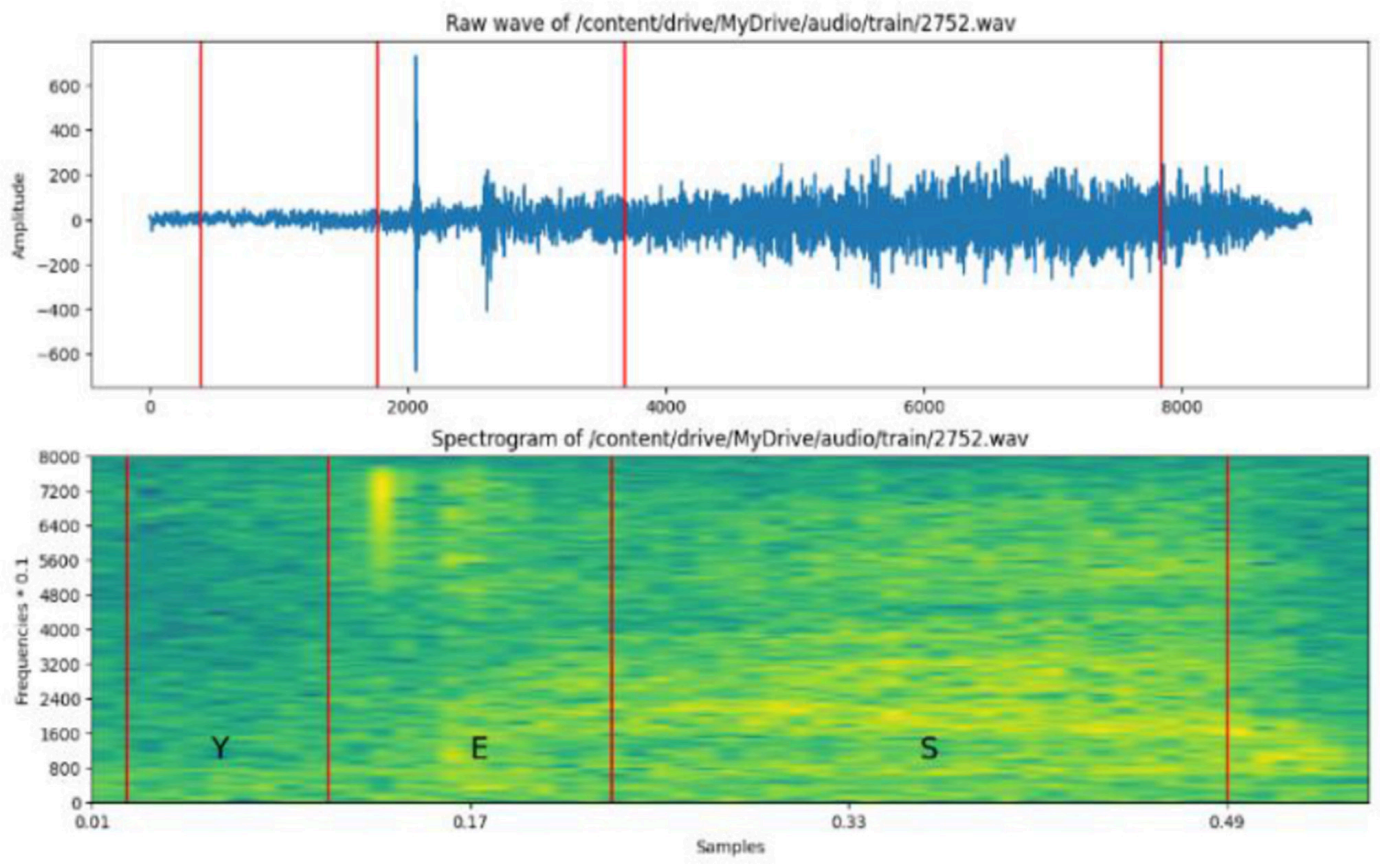
Raw and spectrogram of wave signals.

Figure 3 shows the signal after noise removal analysis of an Arabic signal. Arabic audio signals must be stripped of silent or low-energy segments by identifying and removing them. The advantages of silence removal include speech analysis for cleared content and improved speech clarity.

#### 3.4.2 Time and frequency analysis of speech

The basic frequency of the vocal cords, which determines whether a voice is perceived as high or low, is referred to as pitch. Rapid alterations in the speech signals linked to consonants and other non-voiced sounds are known as transient features. The time-frequency distribution of the signal is mentioned as the frequency spectrum of the audio signal. The specific characteristics of the spectrum will depend on the speaker's voice, the content of the speech, and the recording conditions. Analyzing spectra gains valuable insights into the acoustic properties of speech signals and is helpful for speech recognition, speaker identification, and language understanding.

### 3.5 Sampling

**Figure 4:**
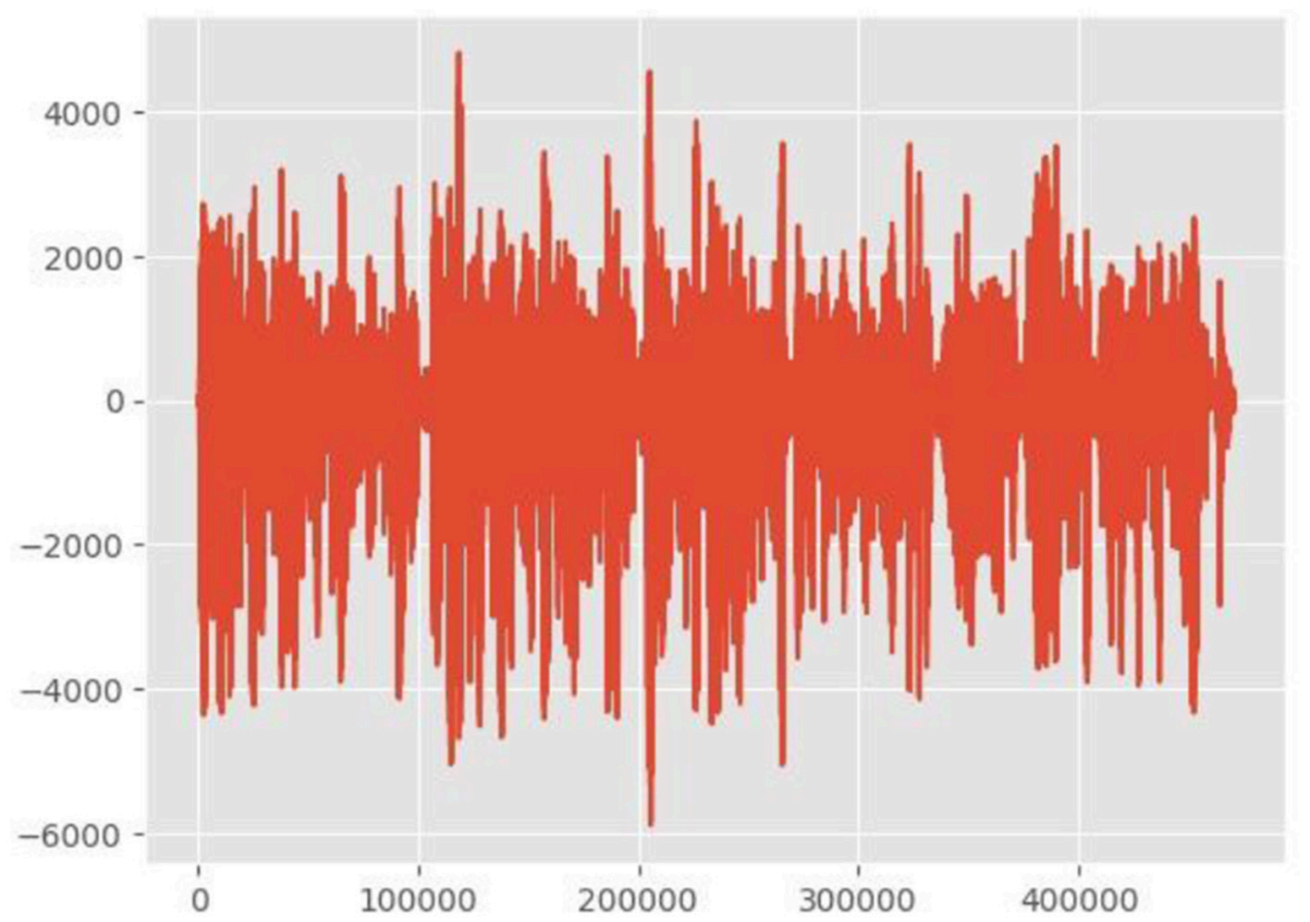
Sampling frame of an audio signal.

Digitalizing the continuous sound wave is necessary for audio signal sampling. We have digitized the sound wave for Arabic audio. To achieve this, the parameters of the sampling rate should be established to determine the frequency of signal measurement. We have used a sampling rate of 44.1 kHz and a bit depth of 16 bits for our Arabic speech for sampling one lengthy audio wave. The overall sampling rate is 16 kHz. Figure 4 shows the sampling frame of the audio signal. Spectra used horizontal and vertical axes to visually represent the energy distribution across time and frequency, respectively. The power of each combination is indicated by the intensity of the color. Common observations include darker areas, which are associated with high energy, and lighter areas, often linked to unvoiced sounds.

#### 3.5.1 Discrete Fourier Transform

**Figure 5:**
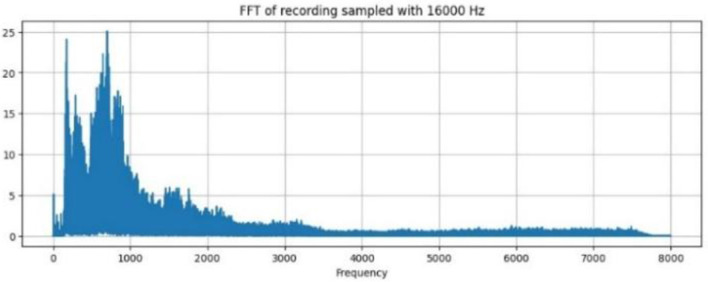
FFT recordings of wave.

The windowed speech signal is subjected to DFT, which yields the signal's phase and magnitude representation. The Fast Fourier Transform (FFT) algorithm transforms time domain analysis to frequency domain analysis Figure 5 shows the FFT spectrum of an audio signal and the distribution of the energy that occurs at different frequencies for each segment. Dominant frequencies are those that indicate prominent tones, such as formants and pitch. The spectral content is used to reveal the presence of various frequency components. The sampling frequency of 1,600 Hz provides basic frequency analysis.

#### 3.5.2 MFCC feature extractions

The process of extracting MFCC features is essential for comprehending speech content, which involves triangular filters. Standard FFTs linearly analyze frequencies of sound, but human hearing operates on a Mel scale. The output of the FFT is passed through triangle-shaped filters. We can capture the portions of the spectrum most pertinent to human hearing by adding the contributions of each filter, each of which focuses on a particular frequency range. The MFCC is the result of this Mel-focused representation. Filters are arranged logarithmically, except above 1,000 Hz, and are equally distributed. The equation used to compute Mel frequency is given in Equation 5 (Gupta et al., 2013).


(5) $$\begin{array}{l} \text{Mel}\left(f\right)=1127\text{In}\left(1+\frac{f}{700}\right) \end{array}$$

**Figure 6:**
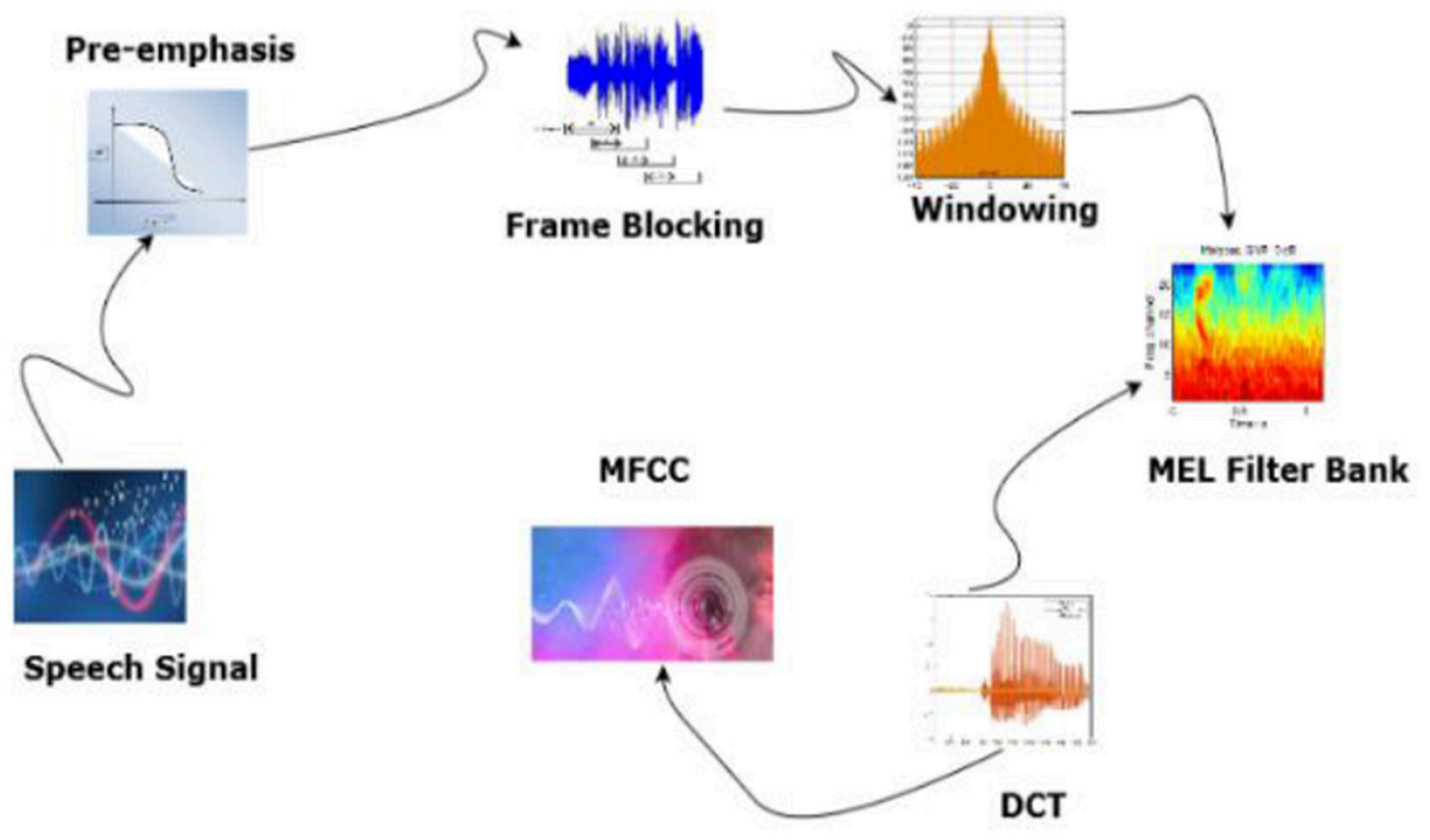
MFCC feature extraction.

The changes in the speech from frame to frame can be calculated with the first and second MFCC coefficients. Figure 6 shows the block diagram of MFCC feature Extraction.

**Figure 7:**
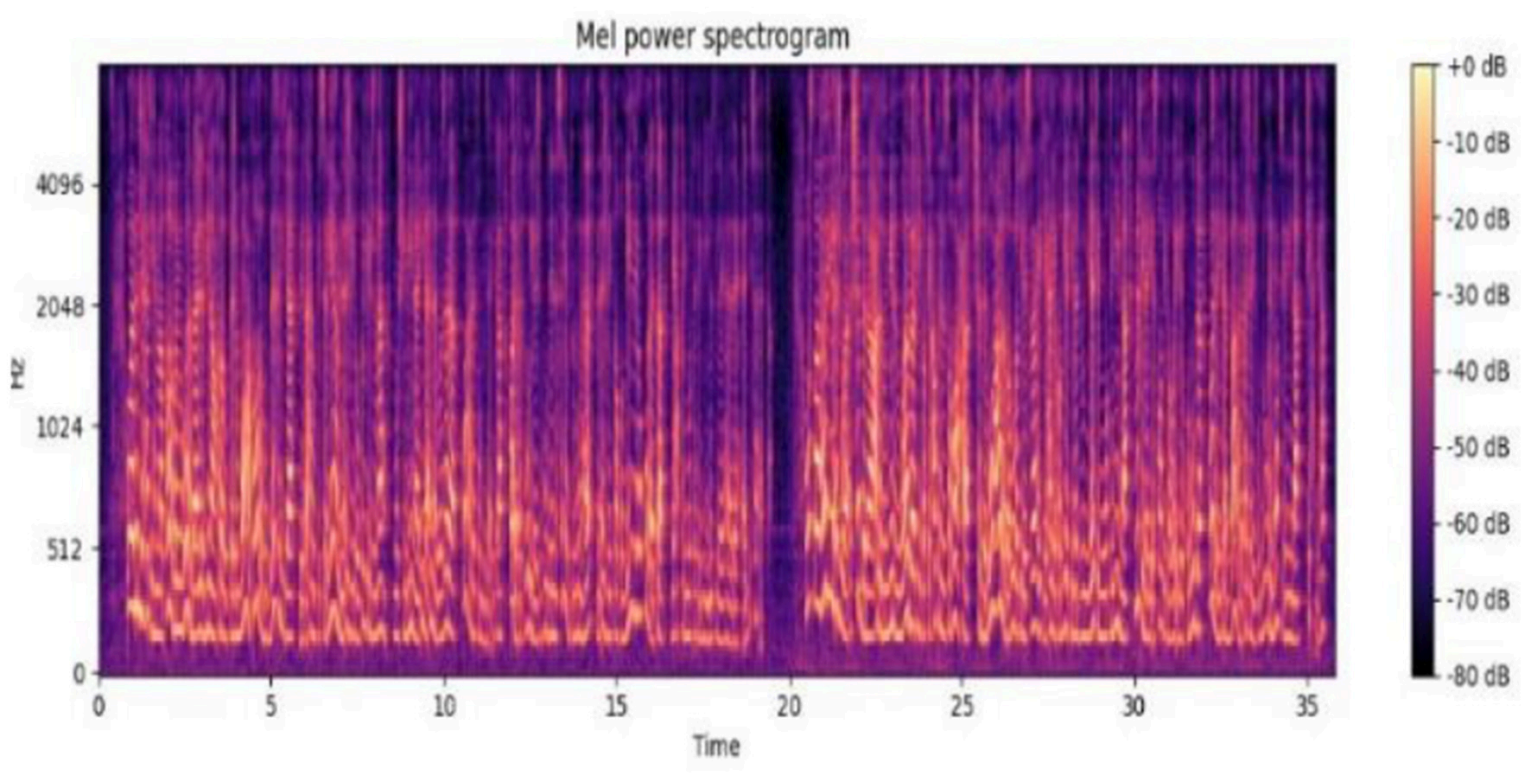
Mel spectrogram.

The audio signal is divided into frames. Windowing and FFT are applied to convert it to the frequency domain. Mel-scale filtering is used in accordance with human auditory perception and logarithmic compression. The discrete Cosine Transform is used to reduce dimensionality, and the resulting MFCCs can provide speaker independence, robustness against noise, and can be processed efficiently. They also capture the fundamental spectral characteristics of speech. Figure 7 shows the Mel power spectrum of the Arabic audio dataset.

#### 3.5.3 MFCC statistics

The mean, standard deviation, maximum, and minimum values are represented in Table 1. The mean reveals the average emphasis on the frequency band within the speech. The speech data's standard deviation is a measure of its variability. The maximum and minimum values help in locating anomalies or errors made during the MFCC extraction process. A Discrete Cosine Transform is applied to each MEL filter band to extract MFCCs from the Mel spectrum.

**Table 1:** MFCC statistics.

**Mean**	**Standard deviation**	**Maximum**	**Minimum**
−52.965	8.573	−19.167	−88.341

**Figure 8:**
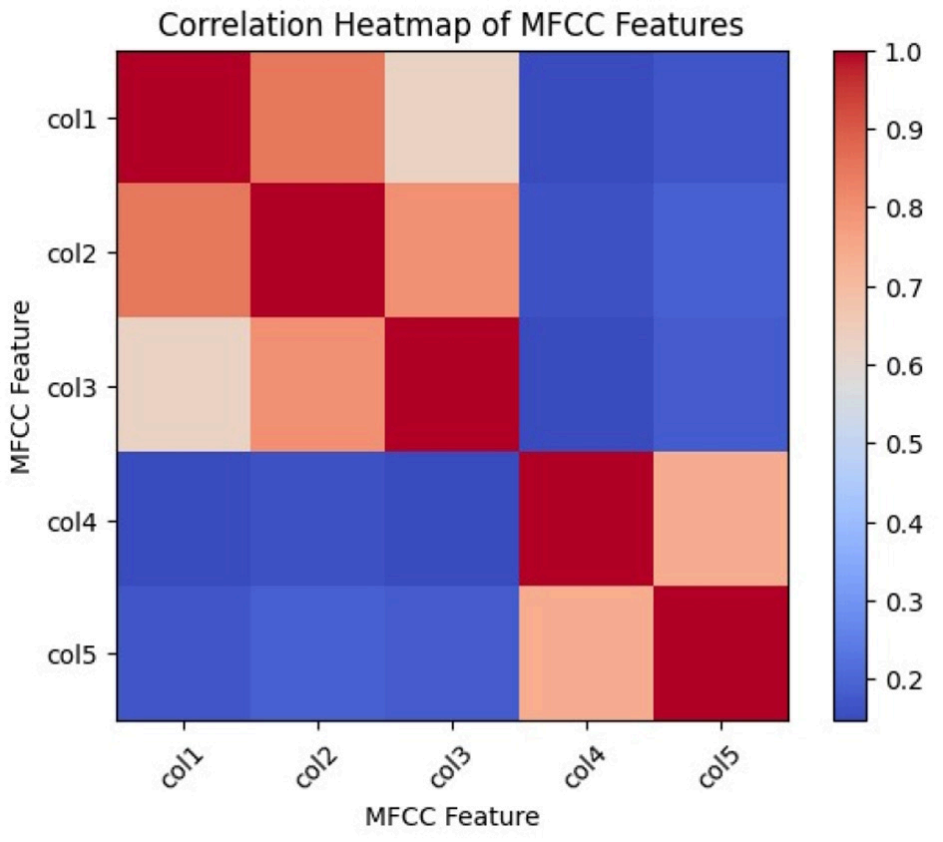
Correlation heat map.

Figure 8 shows the correlation heat map of the different Mel frequency coefficients. The degree of similarity between different MFCCs is shown by their correlation. The various MFCC features are represented by the rows and columns in the heatmap. The correlation between the features that correspond to the row and column is represented by the color of each cell. When two features have a positive correlation, that is, when they tend to rise or fall together, they are colored red. When two features are negatively correlated, one tends to increase while the other decreases, as indicated by blue. When the two features are uncorrelated, the color white is used. Every value on the heatmap's diagonal is 1.0, indicating that every feature has a perfect correlation with every other feature. Higher values indicate stronger correlations. The values of the diagonal range from −1.0 to 1.0. MFCC captures the spectral envelope of audio signals based on the relative prominence of different frequency bands.

## 4 Clustering and classification

**Figure 9:**
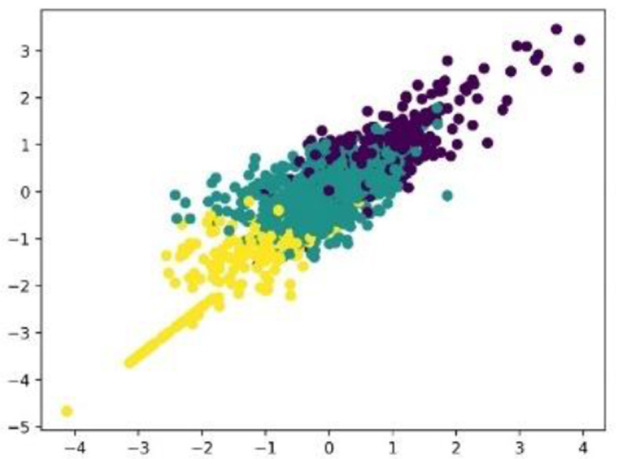
Clustering of MFCC features.

MFCC features are clustered together using a clustering algorithm. As the labels are unknown to us, supervised learning is not a solution to the problem. An unsupervised learning method called K-means clustering will be used for grouping into clusters. The clustering divides data points into a fixed number of groups (K) based on their similarity. The first K data points are chosen at random to serve as the initial cluster centers. The nearest center is determined by averaging these assigned points. Repeating this process until the centers stabilize produces groups in which the data points are unique from those in other clusters and similar to each other within each cluster. Clustering is done based on the Euclidean distance in the MFCC feature space between data points. Three clusters are applied to MFCC features. The clustered data are scaled with a silhouette score. Figure 9 shows the three groups of clusters formed from MFCC correlation features. A silhouette score of 0.6918 was obtained in the clustering. The silhouette score is the metric used to assess the quality of clustering algorithms. It evaluates how well data points are assigned to their clusters. Scores range from −1 to 1, with values closer to 1 indicating improved clustering.

### 4.1 Grid search

In machine learning, grid search is a technique used to determine a model's optimal settings, also known as hyperparameters. Each hyperparameter has a specific range, and the model is trained using all possible combinations from the different ranges. The performance of each combination is assessed, and the best combination is selected as an ideal set. Grid search CV finds the optimal solution based on the selected metric.

### 4.2 Classification

For multiclass classification tasks, the support vector machine classifier is used. A hyperparameter tuning method called grid search is used to maximize the performance of the SVM model. “Linear” and “rbf” for kernel and (Mohammed Ameen and Abdulrahman Kadhim, 2023; Belinkov et al., 2019) for C are the possible values that are explored for the two hyperparameters, “kernel” and “C.” The training data are fitted to the SVM model that performs the best. Confusion matrix and classification report metrics are used in performance evaluation.

## 5 Baidu's deep speech

**Figure 10:**
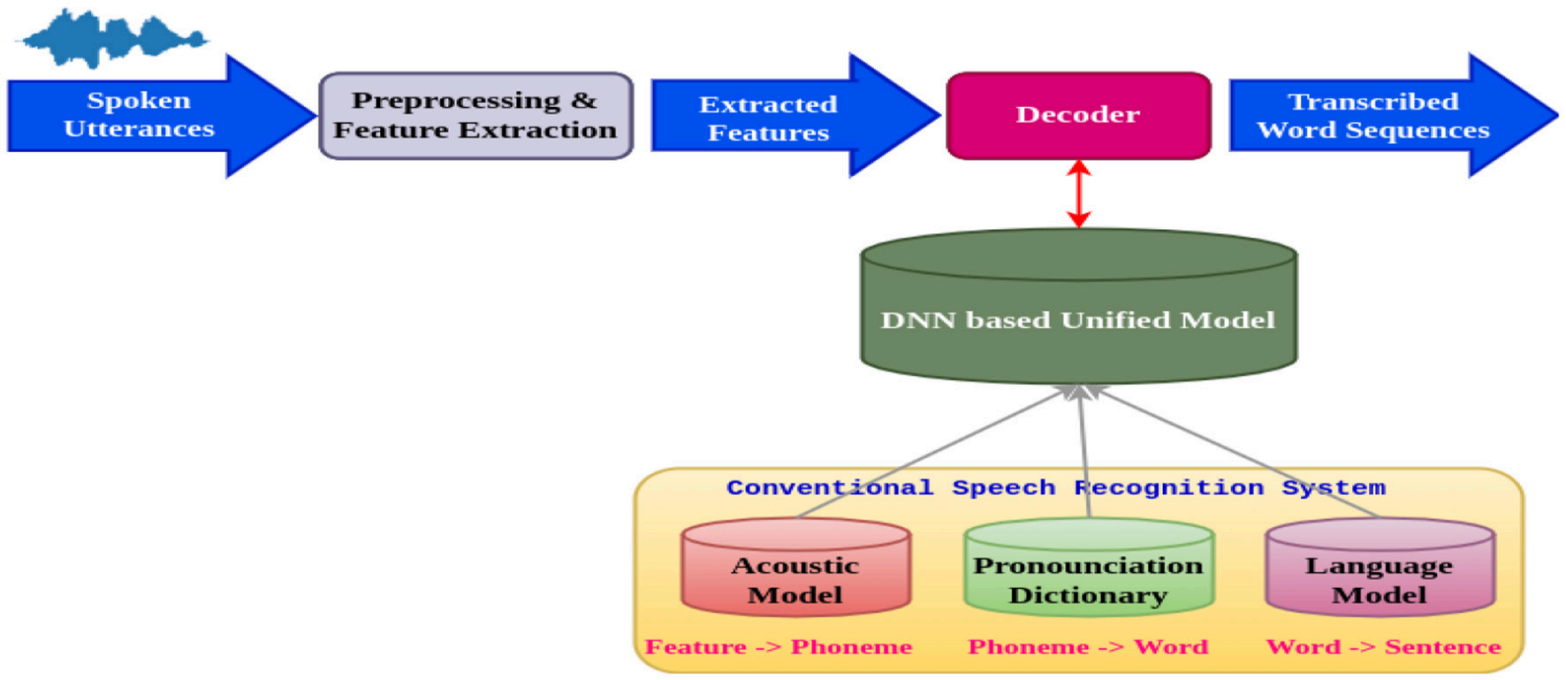
Baidu's Deep speech structure.

The state-of-the-art speech recognition system known as Deep Speech was developed using Baidu's end-to-end ASR architecture. A massive amount of speech data is trained using multiple GPUs and an RNN. Baidu's Deep Speech can learn directly from a large set of data, so it does not require speech adaptation or noise filtering. Deep RNN training will be based on supervised learning. From voice samples, mel-frequency cepstral coefficients are extracted, and transcription is output directly. A full voice recognition system powered by deep learning and its structure. The system generates a matrix of character probabilities, which shows that it gives each character in the alphabet a chance at each period step, indicating the likelihood that that particular character will match the audio. Furthermore, the Connectionist Temporal Classification (CTC) loss function increases the probability of accurate transcription. TensorFlow uses Baidu's Deep Speech Architecture to implement Mozilla Deep Speech, enabling the creation of prototypes for any dialect. It is simpler to operate and performs better in noisy environments than other traditional systems. This system's main advantage is that it outperforms traditional speech recognition systems, capable of handling speaker oscillation, echo, and background noise. From audio files, a time series spectrogram is produced, with each time slice representing a vector of audio characteristics. Three of the five unseen layers that comprise the RNN that powers the Deep Speech model are non-recurrent. Figure 10 shows the architecture of Baidu's Deep Speech system.

### 5.1 Acoustic model and language model

The acoustic archetypal generates a likelihood distribution over the characters of the alphabet in response to audio. The acoustic model takes up the majority of the training time. Typically, three steps are involved in the feature extraction process. The acoustic front end, also known as speech analysis, is the initial phase. It creates raw features by performing a type of temporal analysis of the signal's spectrum. The acoustic model's task is to use the sequence-to-sequence Deep Speech algorithm to identify which acoustic signals correspond to which specific letters. The language model helps translate these probabilities into comprehensible language words, followed by extensive labeled voice training on a large volume of data. The most important things to consider are the data that are rarely or never present in our training sets. We combine our system with one of these n-gram language models since they are readily trained from large unlabeled text datasets. Language models are typically trained by minimizing confusion on training data and by observing word sequences in text corpora that contain millions of word tokens. A variety of toolkits, including SRILM, KENLM, and open-game toolkits, are used to generate language models. It is necessary to train the linguistic model and the audio model with the same alphabet. alphabet.txt is the glue that holds the linguistic model and the acoustic model together. The neural network utilized in the acoustic model was trained on a corpus of voice and transcripts, which was created with TensorFlow. An n-gram model trained with KENLM is the morphological ideal, and the training data are a corpus of text. As inputs are fed into the network for a reference window of size k, the ith unit in a convolutional layer l at a timestamp t delivers M(l,i), as shown in Equation 6, which represents the architecture of a deep RNN using Arabic data.


(6) $$\begin{array}{l} M^{\left(l,i\right)}=\sigma \left(\omega^{\left(l,i\right)}\cdot M_{t-k:t+k}^{l-1}\right) \end{array}$$

Here, M(0) denotes the input, and it contains 13 units. σ(.) is the activation function as in Equation 7, and the hidden fully connected layers use a Rectified Linear Unit (ReLU) activation function. We always constrain the output of a convolution unit to up to 5 (Wu et al., 2024).


(7) $$\begin{array}{l} \sigma \left(x\right)=\min\left(\max\left(0,x\right),5\right) \end{array}$$

At any timestamp *t*, the units at layer l of the recurrent bidirectional LSTM take updates from both past and future timestamps, as shown in Equations 8, 9.


(8) $$\begin{array}{l} \overset{\to }{M_{t}^{l}}=tanh\left(\omega^{l}\cdot \text{M}+\overset{\to }{U^{l}}\cdot \overset{\to }{M_{t-1}^{l}}+b^{l}\right) \end{array}$$

(9) $$\begin{array}{l} \overset{\leftarrow }{M_{t}^{l}}=tanh\left(\omega^{l}\cdot \text{M}+\overset{\leftarrow }{U^{l}}\cdot \overset{\leftarrow }{M_{t+1}^{l}}+b^{l}\right) \end{array}$$

where ω^*l*^ is the input hidden weight matrix and *U*^*l*^ is a recurrent weight matrix. The sum of forward and backward directional states yields an “informed state” (hl), which is shaped by the prior transitional probabilities of the phonemes. The activation function tanh(.) acts like a squashing function, as shown in Equation 10 (Morais, 2025).


(10) $$\begin{array}{l} \tan h\left(x\right)=\frac{e^{x}-e^{x}\;}{e^{x}+e^{x}} \end{array}$$

The processed cepstral coefficients flow through the recurrent layers, and each upper layer receives this processed information from its immediate lower layer, which is given in Equation 11.


(11) $$\begin{array}{l} M_{t}^{l}=f\left(\omega^{l}\cdot M_{t}^{l-1}+b^{l}\right) \end{array}$$

The output is a softmax layer that gives a probability distribution over phonemes, shown in Equation 12.


(12) $$\begin{array}{l} P\left(o_{t}^{k}=k/x\right)=\frac{e^{\omega_{k}^{L}\cdot h_{t}^{L-1}}}{\Sigma_{i}e^{\omega_{k}^{L}\cdot h_{t}^{L-1}}} \end{array}$$

**Figure 11:**
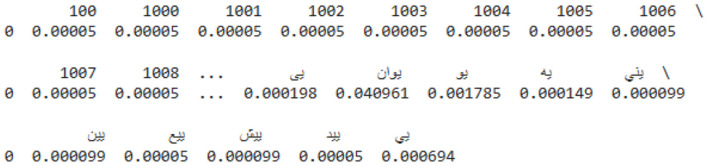
Sample TF-IDF vectorizer data.

The value of the output unit at any timestamp *t* will indicate the probability of the corresponding phoneme n as predicted by the network. The network is then trained using the CTC loss function, and the parameters of the network are updated using the backpropagation through time (BPTT) algorithm. Then 32-bit beam search decoder is used to construct the output from the phoneme distribution. The Term Frequency Inverse Document Frequency (TF-IDF) vectorizer is a useful tool for translating Arabic text data into numerical vectors. When analyzing text at the character level, it considers individual characters, pairs of characters, and triplets of characters. This is an important step for the Arabic script. It learns the vocabulary and term importance from the data and then creates TF-IDF vectors for each document. Based on the frequency of each term in the document and rarity across the dataset, these vectors indicate the relative importance of each term. Then, among other NLP tasks, these vectors can be used to train machine learning models for document classification, hidden topic identification, and document similarity comparison. The two main tasks completed by the vectorizer are stemming/lemmatizing Arabic text and normalizing it. The sample data are shown in Figure 11.

**Figure 12:**
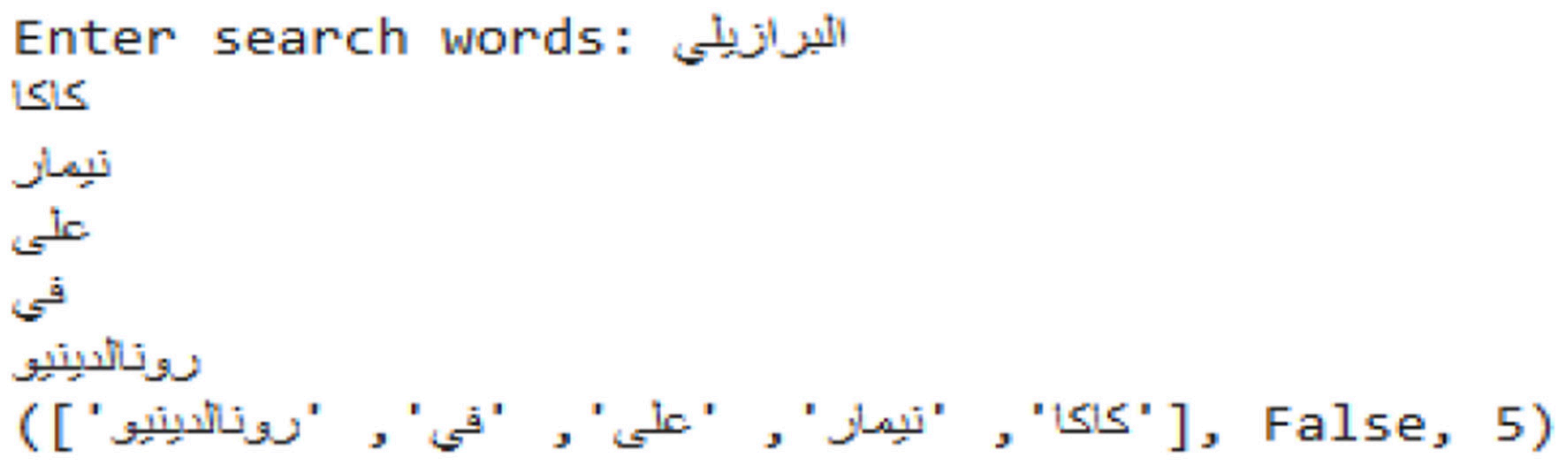
Sample n-gram prediction.

To calculate the probability of each sentence, the function counts the number of sentences (n-grams) that have been viewed so far, divides that count by the total number of sentences, and increases the count for each sentence. This is a basic method to determine the word or words that will appear next in a given sequence and to calculate the probability that a sentence will appear again based on how frequently it appears in the dataset. It separates Arabic text data into words, cleans it up, and calculates the probability that different word combinations (n-grams) will occur together. A sample prediction is shown in Figure 12.

### 5.2 Augmentation and hyperparameter setup

#### 5.2.1 Baidu's deep speech hyperparameters

The majority of the hyperparameters in the preconfiguration for Mozilla Deep Speech remained unchanged. Nonetheless, the batch size was slightly modified in consideration of the machine's capabilities and the amount of training data. Furthermore, Deep Speech offers the ability to create checkpoints, allowing training to be resumed in the event of an error using the checkpoints. Either we create a checkpoint directory and store the training checkpoints there, or we freight the Deep Speech frontier directory containing the training checkpoints. Prediction accuracy is calculated using the loss. As the loss decreases, the difference between the neural network's predictions and the actual known values becomes smaller. When there is no reduction in loss, the parameter indicates how many training epochs should be considered as a plateau.

- **Hyperparameter optimization:** Optuna is a framework utilized for hyperparameter optimization. It specifically adjusts lm_alpha, which is a language model weight, and lm_beta is a word insertion bonus. To reduce the WER and CER on a designated test set, it systematically assesses several combinations of these parameters, dynamically reinitializing the TensorFlow graph for each iteration and relaying intermediate performance metrics to Optuna, which subsequently directs the search intelligently and eliminates unpromising trials to enhance efficiency. The model ascertains whether to optimize for WER or CER according to the loaded scorer's mode and offers a definitive entry point for users to commence this essential post-training optimization procedure, yielding the optimal parameters and their associated performance.
- **Reduce plateau**: If training does not result in a decrease in loss over time, it is said to have plateaued. It is possible to break through the plateau and keep reducing losses by adjusting the learning rate and other parameters.
- **Early stopping:** If training does not eventually reduce loss, an early termination is an option.
- **Dropout:** When training produces a model with poor generalization, it is referred to as overfitting and has an impact on the model's generalizability. A method called “dropout” enhances the generalizability of the model by arbitrarily eliminating nodes from the neural network to lessen overfitting.
- **Steps and Epochs:** A training set's entire cycle is referred to as an epoch. Batch size affects how much memory is required for processing. Fifteen epochs and a batch size of four are employed for this optimization.
- **Train–test split:** The training loop efficiently manages data loading, preprocessing, and augmentation, while enabling multi-GPU training by distributing computations across “towers” to average gradients for faster updates. Key components, including adaptive learning rate reduction during performance plateaus, early stopping to prevent overfitting, and thorough checkpointing, which entails retaining the best-performing model on a validation set, are integrated to ensure rapid and effective model development. This provides functionalities for autonomous evaluation of models on test datasets and the creation of efficient inference graphs, representing a complete solution for DeepSpeech model training and deployment. We have utilized 70% of the audio data for training 15% for testing, and 15% for validation.

#### 5.2.2 Machine learning hyperparameters

Table 2 shows that the grid search method uses different values of hyperparameters in each run. The first run uses the C values of 73, 79, 50, and 52, while the second run uses the C values of 19, 81, 72, and 89. The fit and score time are mentioned in Table 2.

**Table 2:** Hyperparameters of grid search.

**Scores**	**Decision tree**	**XGBoost**	**KNN**	**Random forest**
Mean fit time	0.0135	0.0317	0.0234	0.0293
Standard fit time	0.0007	0.0009	0.0020	0.0009
Mean score time	0.0037	0.0112	0.0030	0.0101
Standard score time	1.2655	4.6037	7.41052	1.0215
Mean test score	0.9973	0.9886	0.9980	0.9900
Standard test score	0.0020	0.0028	0.0019	0.0027
Rank test score	2.000	3.000	1.000	3.000

#### 5.2.3 Computational environment

All experimental methods were performed on a MacBook Pro, specifically configured with a 1.4 GHz Quad-Core Intel Core i5 processor. The system employed Intel Iris Plus Graphics 645 for graphics processing, featuring 1,536 MB of memory. The device was equipped with 8 GB of 2,133 MHz LPDDR3 RAM and ran macOS Sequoia version 15.5. The dataset and computational outputs were stored on a 250.69 GB Macintosh HD, with 112.16 GB of space available during the experimental phase. This configuration facilitated the computational framework for all data processing, model training, and evaluation activities conducted in this research.

## 6 Results and discussions

### 6.1 Confusion matrix

**Figure 13:**
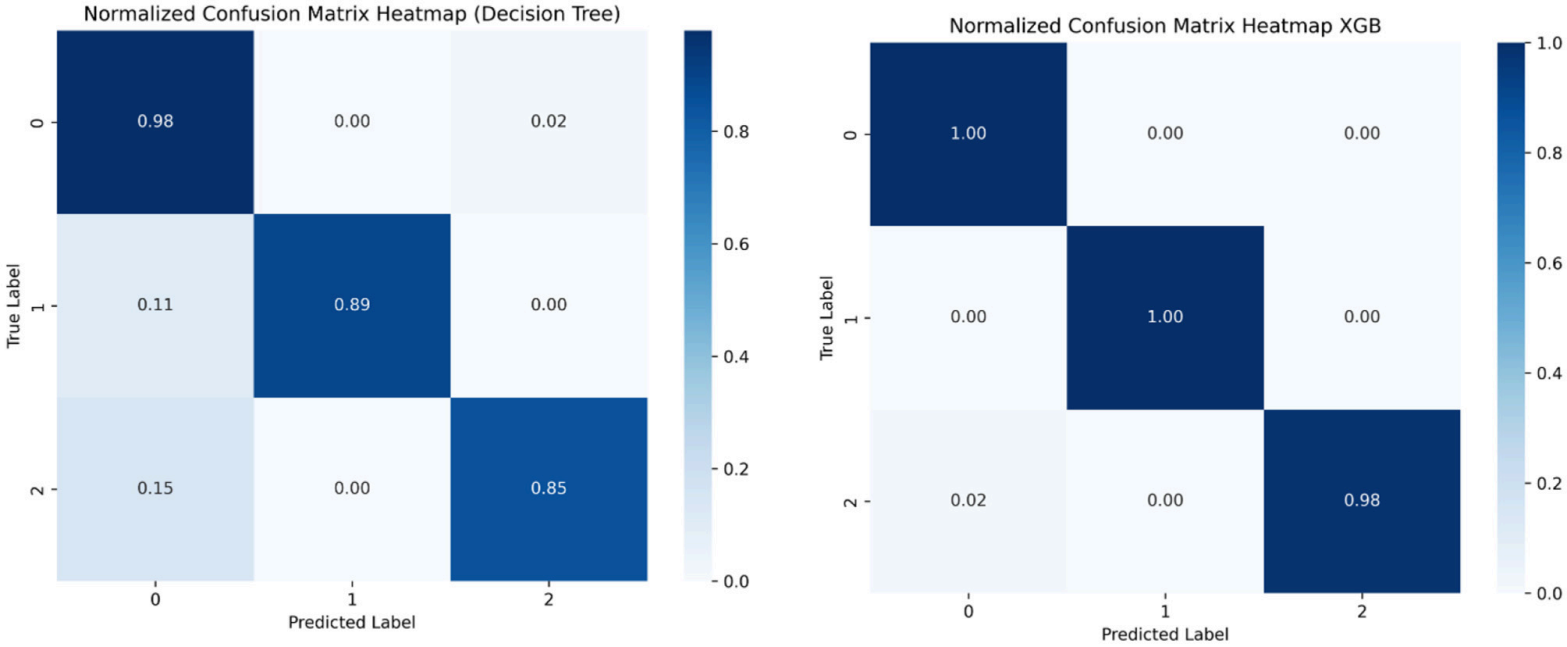
Confusion matrix, decision tree, and XGBoost.

**Figure 14:**
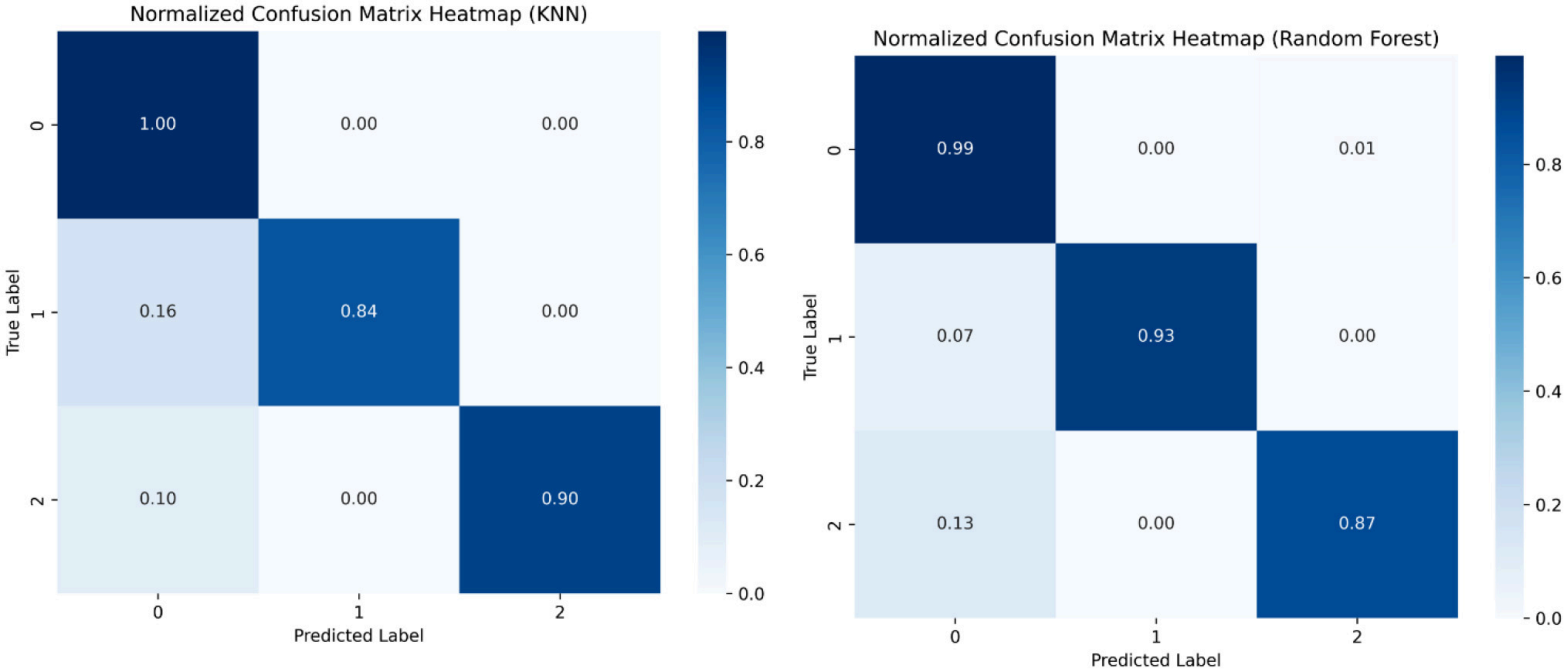
Confusion matrix KNN and Random Forest.

Confusion matrices are specially used to visualize a model's performance in classification problems. They display the frequency of errors, such as false positives and false negatives, as well as the proportion of correctly classified data points, such as true positives and true negatives. The model predicts 1,145 actual instances of class 1 correctly and 55 actual instances of class 2, and 86 out of 87 actual instances of class 3. Figures 13, 14 show the confusion matrices.

### 6.2 Classification report

Both the confusion matrix and classification report indicate that the model achieved excellent performance with perfect accuracy, precision, recall, and F1-score for each class. Table 3 shows the classification report.

**Table 3:** Classification report.

**Classifiers**	**Class**	**Precision**	**Recall**	**F1-score**	**Support**
Decision tree	0	1.00	0.99	0.99	99
	1	1.00	1.00	1.00	1134
	2	1.00	1.00	1.00	54
XGBoost	0	0.99	0.98	0.98	99
	1	1.0	1.0	1.0	1126
	2	1.00	0.98	0.99	62
KNN	0	0.95	0.87	0.91	95
	1	0.98	1.00	0.99	1137
	2	1.00	0.76	0.87	55
Random Forest	0	0.88	0.90	0.89	78
	1	0.99	0.99	0.99	1153
	2	0.98	0.95	0.96	56

### 6.3 Learning curve

**Figure 15:**
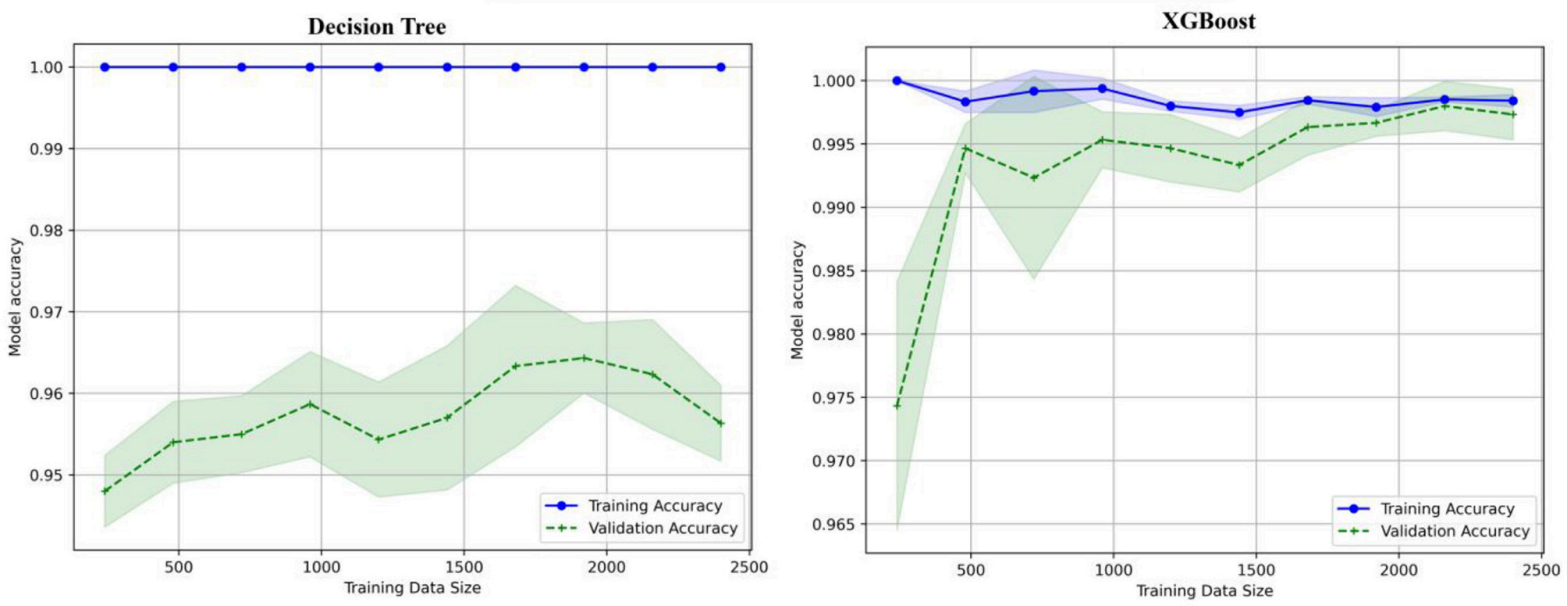
Learning curve for decision tree and XGBoost.

**Figure 16:**
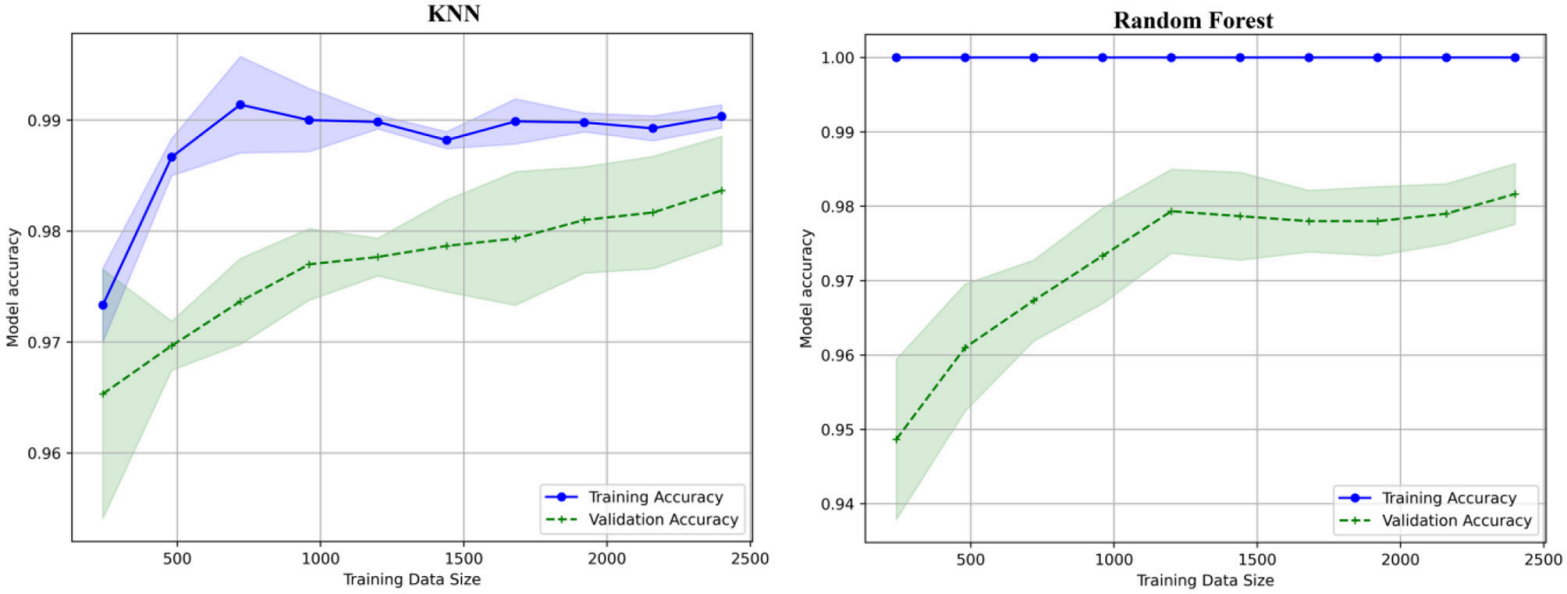
Learning curves for KNN and random forest.

The learning curve shows the x-axis with values between 500 and 2,500 labeled as training data size, shown in Figure 15. The model accuracy y-axis has a range of 0.95 to 1.0. Two lines are displayed, one green for validation accuracy and one blue for training accuracy. As the size of the training data increases, the validation accuracy also increases, indicating that data are being trained well and validated. The learning curves for the decision tree, XGboost, KNN, and Random Forest are shown in Figures 15, 16.

### 6.4 Precision–recall curve

**Figure 17:**
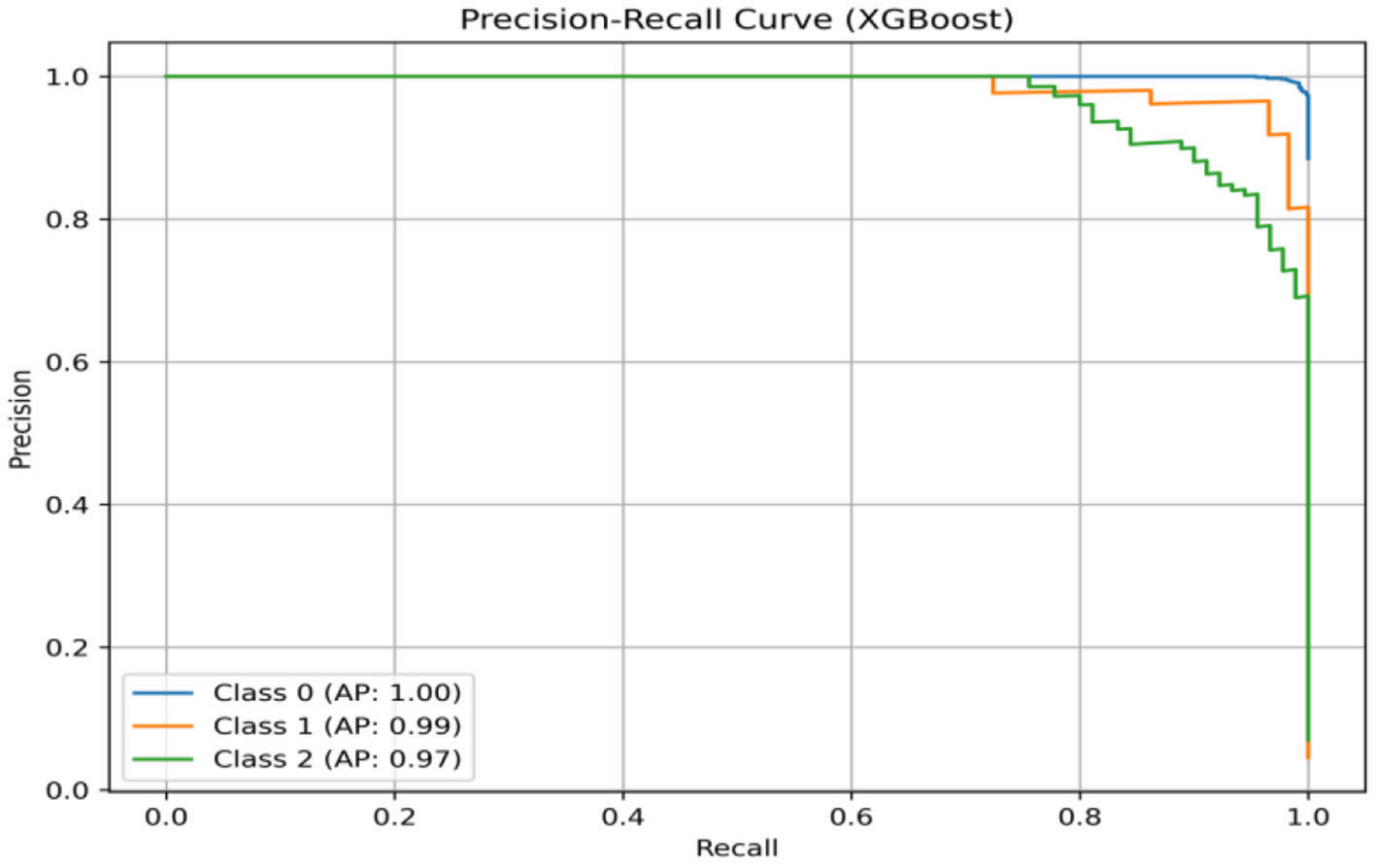
Precision–recall curve for XGBoost.

The graphical tool called a precision–recall curve (PRC) is used to assess how well the classification model performs in multiclass problems, as shown in Figure 17. PRCs offer insight into the tradeoff between precision and recall in contrast with the receiver operating characteristic area under the curve (ROC AUC), which concentrates on binary classification. The ROC AUC score is obtained as 0.99928. The WER is the percentage of words that the system incorrectly recognizes, and the CER is the percentage of characters that the system recognizes incorrectly. This shows that the speaker's ability to speak correctly has improved, as has the speech recognition system's ability to recognize their speech. The graph also shows that the WER continuously outperforms the CER. This is because the speech recognition system finds it easier to identify individual characters.

**Figure 18:**
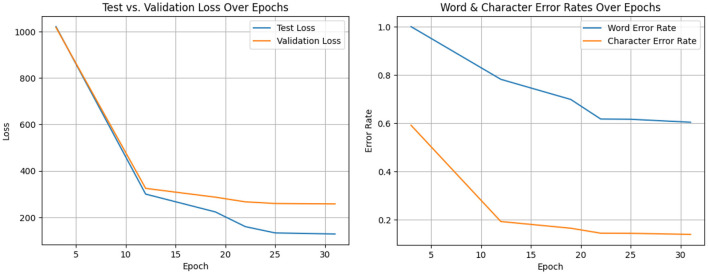
Test and validation loss vs. epochs and word and character error rate vs. epochs.

Figure 18 shows the test and validation loss vs. various epochs and the word and character error rate vs. epochs of the system's WER and CER plotted against time. The WER is the percentage of words that the system incorrectly predicts, and the CER is the percentage of characters that the system incorrectly predicts (Baghdasaryan, 2022). The graph shows that both the WER and CER show a decrease over time, suggesting that the system's speech recognition performance is improving. In contrast, the WER constantly exceeds the CER. The reason for this is that individual characters are recognized by the algorithm more readily than entire words. The graph also shows how the WER and CER start to plateau after a certain number of epochs. The graph shows that the voice recognition system is training effectively. The system's increasing efficiency is demonstrated by the decrease in WER and CER over time. The word error rate is the most popular metric for ASR.


(13) $$\begin{array}{l} \text{WER}=\frac{S_{w}+D_{w}+I_{w}}{N_{w}} \end{array}$$

When a word in the reference sequence is transcribed as a different word, it is called a substitute word (Sw). When a word is completely absent from the automatic transcription, it is referred to as a deleted word (Dw). The number of words inserted is Iw. This means the word's appearance in the transcription has no correspondent in the reference word sequence. As it lacks the upper bound, the word error rate only indicates whether one system is superior to another. For this reason, a character error rate is used.


(14) $$\begin{array}{l} \text{CER}=\frac{s+d+i}{N} \end{array}$$

Table 4 describes the entire model analysis. The size and complexity of the exercise data, along with the system's design, will determine the ideal number of epochs for training a speech recognition system.

**Table 4:** Model performance analysis.

**Epoch**	**Test loss**	**Validation loss**	**Word error rate**	**Character error rate**
3	1,017.0	1021.4	1.0000	0.59118
12	300.00	324.70	0.7815	0.1920
19	223.27	286.77	0.6982	0.1643
22	160.01	266.72	0.6170	0.1437
25	132.86	259.57	0.6160	0.1432
31	128.33	257.66	0.6037	0.1387

Table 5 illustrates the best model analysis and the corresponding transcribed Arabic text.

**Table 5:** Model performance analysis—best model.

**Epoch**	**Best WER**	**Best CER**	**Loss at best WER/CER**	**Arabic text**	**English text**
12	0.4687	0.1060	110.289	السِّيفقِصَرِفِيالأُسبُوعِيّاِجتِمَاعُهُالوُزَرَاءِمَجلِسُعَقَدَ المحَمَّدنَاصِرالشِّيخالشِّيخمَجلِسِرَئِيسِسُمُوِّبِرِئَاسَةِ كَشَفَالهَامَّةالمِلَفَّاتِمِنمَجمُوعَةٍالوُزَرَاءُتَدَاوَلَتحَيثُ رَوضَانِالالوُزَرَاءمَجلِسِلِشُؤُونِالدَّولَةوَزِيرِعَنهَا الرَّوضَان	The Cabinet held its weekly meeting at Seif Palace under the chairmanship of His Highness the Prime Minister Sheikh Nasser Al-Mohammed, where the ministers deliberated a set of important files revealed by Minister of State for Cabinet Affairs Roudhan Al-Roudhan
19	0.3720	0.0568	276.147	بَعدَأَشهُرمُنذُإِضرَابَاتٍيَشهَدُاليَمَنأَنَّإِلَىالإِشَارَةُتَجدُرُ وَتِلكَالحَاكِملِلنِّظَامِالمُؤَيِّدَةِوَالمَسِيرَاتِالمُظَاهَرَاتِ عَلِيِّالرَّئِيسيَتَلَقَّىفِيمَابِإِسقَاطِهتُطَالِبُوَالَّتِيلَهالمُعَارَضَةِ عَقِبَالسَّعُودِيَّةالسَّعُودِيَّةالمَملَكَةِفِيالعِلَاجَصَالِحعَبدالله الشَّهرهَذَامِنسَابِقٍوَقتٍفِيالرِّئَاسِيّالقَصرِعَلَىهُجُومٍ	Yemen has been witnessing strikes for months after demonstrations and marches in support of the ruling regime and those opposing it, demanding its ouster, while President Ali Abdullah Saleh is receiving treatment in Saudi Arabia following an attack on the presidential palace earlier this month.

### 6.5 Discussion

Upon examining the performance of diverse ASR models, some significant themes and insights arise concerning their efficacy across various languages and architectural methodologies. The data reveals a wide range of WERs, from an exceptional 0.720% for the suggested Arabic DeepSpeech model to a maximum of 58.87% for Kazakh utilizing Kaldi. Recent improvements in deep learning models, especially Transformer-based architectures such as XLSR-Wav2Vec 2.0 for Turkish, exhibit markedly lower word error rates (0.23%) compared to previous or toolkit-based methodologies. DeepSpeech is a widely utilized model for several languages (Bengali, Russian, German, Tunisian, Arabic), although its efficacy fluctuates, indicating a significant impact of linguistic attributes and dataset quality. The incorporation of various languages, including Arabic, Bengali, German, Hindi, Kazakh, Russian, Tunisian, and Turkish, emphasizes the international endeavor in ASR development while revealing persistent challenges in attaining universal high performance, particularly for languages characterized by intricate phonetics or scarce resources. The efficacy of the built Baidu's Deep Speech model was meticulously assessed using an independent test dataset in our proposed work. This dataset, completely omitted from the model's training and validation phases, functioned as a vital assessment of the model's capacity to generalize to novel, previously unencountered data. Our results indicate that the model attained a WER of 0.3720 and a CER of 0.0568 during training and 0.19 WER and 0.02 CER during the testing phase.

The unsupervised clustering of MFCC features, together with traditional machine learning classification, could be applied to enhance speaker diarization, acoustic scene categorization, or, importantly, Arabic dialect identification from various audio sources. This feature is essential for augmenting customer service analytics, expanding accessibility tools, facilitating more efficient content filtering, and enriching language learning systems. Furthermore, the framework's proven effectiveness with unlabeled data provides a means for creating ASR solutions for additional low-resource languages or specialized fields that lack comprehensive annotated corpora, thus expanding its influence within the speech technology sector. Table 6 shows the comparison with previous studies.

**Table 6:** Comparison table with previous works.

**Reference**	**Year**	**Model**	**Language**	**WER**
Kazakh speech and recognition methods (Karabaliyev and Kolesnikova, 2024)	2024	Kaldi Mozilla DeepSpeech Google Speech-to-Text API	Kazakh speech	56.87% 55.36% 52.97%
End-to-end Bengali speech recognition (Nahid et al., 2019)	2019	Bidirectional LSTM	Bengali speech	8.20%
Russian-language speech recognition (Iakushkin et al., 2018)	2018	DeepSpeech	Russian speech	18%
German speech recognition (Xu et al., 2020)	2020	DeepSpeech	German speech	12.3%
German end-to-end speech recognition (Agarwal and Zesch, 2019)	2019	DeepSpeech	German speech	15.1%
Tunisian dialectal end-to-end speech recognition (Messaoudi et al., 2021)	2021	DeepSpeech	Tunisian speech	24.4%
Hindi speech recognition (Kumar et al., 2012)	2012	HTK	Hindi speech	12.99%
Transformer-based Turkish automatic speech recognition (Tasar et al., 2024)	2024	XLSR-Wav2Vec 2.0	Turkish Speech	2.3%
Arabic phonic transcription (Elmahdy et al., 2011)	2011	ACA	Arabic	19%
Arabic autoencoder speech recognition (Mohammed Ameen and Abdulrahman Kadhim, 2023)	2023	Deep learning models	Arabic	4%
Convolutional neural networks to facilitate the continuous recognition of Arabic speech (Sayed et al., 2024)	2024	CNN-LSTM	Arabic	3.63%
Arabic speaker-independent continuous automatic speech recognition (Abushariah et al., 2012)	2012	Hidden Markov models	Arabic	11.27%
Proposed study		Baidu's Deep Speech	Arabic Speech	3.7%

## 7 Conclusion

In this study, we examined the effectiveness of using clustering and classification techniques in conjunction with MEL frequency extraction for Arabic audio data processing. This study also briefs on the effectiveness of Baidu's Deep Speech in Automatic speech recognition of the Arabic dataset. Our results demonstrate that MFCCs efficiently capture important features, facilitating the successful clustering of audio segments using K-means or hierarchical clustering algorithms. Additionally, we obtained a low loss of 128.33 for the training dataset and a validation loss of 257.66 by using Baidu's Deep Speech. The WER for the reference is 0.19, indicating that 19% of the words were misidentified. 2% of the characters in the reference were misidentified, according to the CER of 0.02 in the testing phase. The evaluation's findings are encouraging. The model has a respectable level of accuracy regarding Arabic speech recognition.

### 7.1 Future studies

Future studies might investigate applying the existing methods to other widely used Arabic dialects. Potential applications such as assistive technologies for the hearing-impaired, voice-enabled services in Arabic-speaking regions, and integration with NLP pipelines are possible. This would entail developing acoustic models tailored to a particular dialect or investigating transfer learning strategies to modify the current model to accommodate new dialectal data. Also, predicting the next word and character from Arabic text for audio-impaired individuals can be possible from the transcribed data.

## Data Availability

The datasets presented in this article are not readily available due to privacy reasons. Requests to access the datasets should be directed to fawaz.alanzi@ku.edu.kw.
